# Ectopia and Partial Transposition of Mandibular Lateral Incisors in a Child Patient

**DOI:** 10.5402/2011/329067

**Published:** 2010-09-22

**Authors:** Viviane Andrade Cancio de Paula, Felipe Giacomet, Ana Maria Bolognese, Lucianne Cople Maia

**Affiliations:** Department of Pediatric Dentistry and Orthodontics, School of Dentistry, Federal University of Rio de Janeiro, 21941-901 Rio de Janeiro, RJ, Brazil

## Abstract

Dental ectopia is a rare clinical finding characterized by a change in the normal tooth eruption pathway. In more severe cases, nontreated ectopia may develop into either partial or total transposition. The early diagnosis is of crucial importance for establishing a treatment planning correctly. Therefore, the present paper is aimed at reporting an unusual case of a 11-year-old boy with ectopic eruption and partial transposition of mandibular permanent lateral incisors as well as the diagnosis and therapeutic outcomes involving such an anomaly.

## 1. Introduction

Dental ectopia is characterised by the change in the normal pathway of a tooth eruption, which may occur in any region of the alveolar and basal bone [1]. In fact, it is a rare developmental anomaly whose aetiology is unknown and controversial. One can suppose that such an eruption process can be altered by genetic factors [2–8], physical obstacles [9, 10], or multiple causes [3, 10, 11]. It has been demonstrated that dental ectopia is more frequently seen in girls [8, 12, 13]. The occurrence of ectopic eruption is usually unilateral [1, 4, 13, 14], but bilateral cases have been reported [13, 15], and mandibular lateral incisors are the most affected teeth, representing 30% of all cases [16, 17]. 

Ectopic eruption of mandibular permanent lateral incisors can result in both advanced root resorption and precocious exfoliation of the deciduous canines and first molars [1, 15, 17]. Prolonged retention of deciduous canines and lateral incisors can occur as well [4, 10, 15]. Clinically, the ectopically erupted lateral incisor shows marked distal inclination and rotation achieving up to 180° [1, 4, 7, 17, 18]. The diagnosis of such dental anomaly is crucial for establishing the treatment plan and should be carried out through both clinical and radiographic exams, although other exams such as volumetric computerised tomography and study models can be employed [18–20]. If not treated early, this dental anomaly may develop into partial or complete transposition of the permanent canines [7, 18, 20]. 

Therefore, the objective of the present paper is to report a case of male paediatric patient with bilateral ectopia of the mandibular permanent lateral incisors and discuss both implications regarding such an anomaly and treatment outcomes.

## 2. Description of Case

Caucasian male patient of 11 years old was brought by his mother to the paediatric dentistry clinic complaining that the child's two mandibular teeth “appeared to be tilted”.

**Figure 1:**
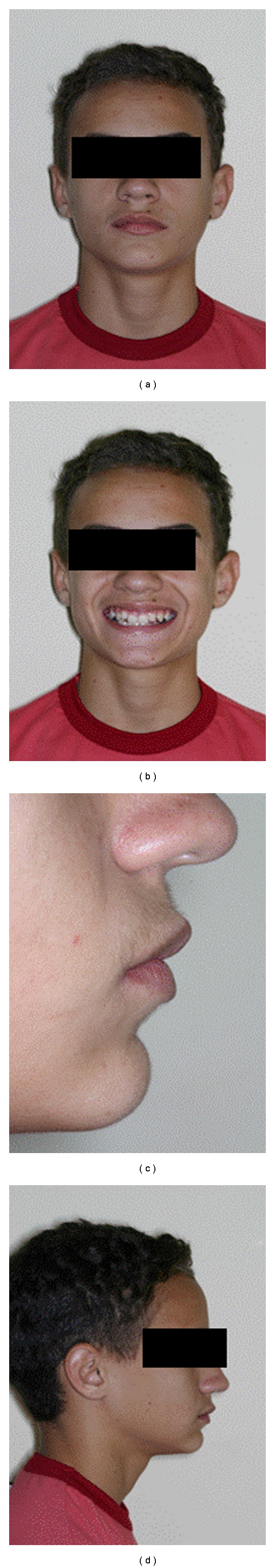


During the interview the mother reported no relevant previous medical history and no cases of ectopia in the family as well. On facial examination, the patient had balanced face with proportional facial thirds and no apparent asymmetry. Convex facial profile and a mild mandibular retrusion were also observed (Figure 1). 

**Figure 2:**
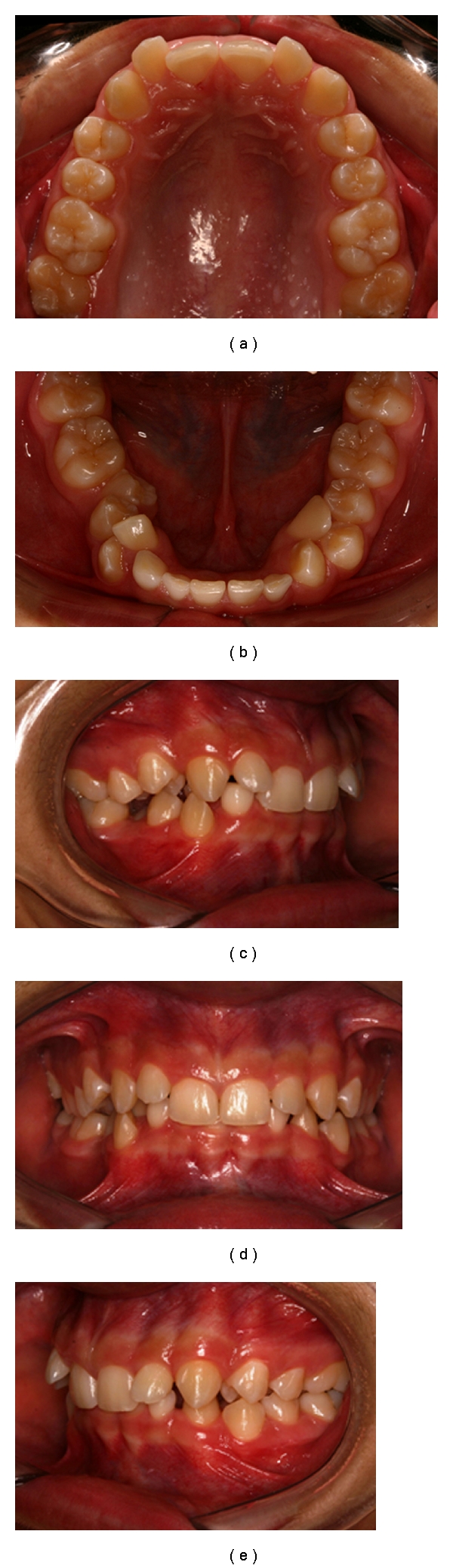


On intraoral examination, it was observed absence of carious lesions, and all the maxillary teeth had been erupted except the third molars whereas permanent right and left lateral incisors were buccally rotated and dislocated. Mandibular arch had all the permanent teeth erupted except the third molars whereas primary left lateral incisor and right lateral incisor and canine had prolonged retention. Permanent right and left lateral incisors were found to be ectopically erupted, with crown transposing the permanent canine on the left side and positioning lingually on the right side; both lateral incisors and canines were rotated and the mandibular right second premolar was impacted. Both maxillary and mandibular dental arches had parabolic shapes with 100% overbite, overjet of 3 mm, and no crossbite. A Class II division 2 subdivision left anteroposterior relationship was diagnosed (Figure 2).

**Figure 3:**
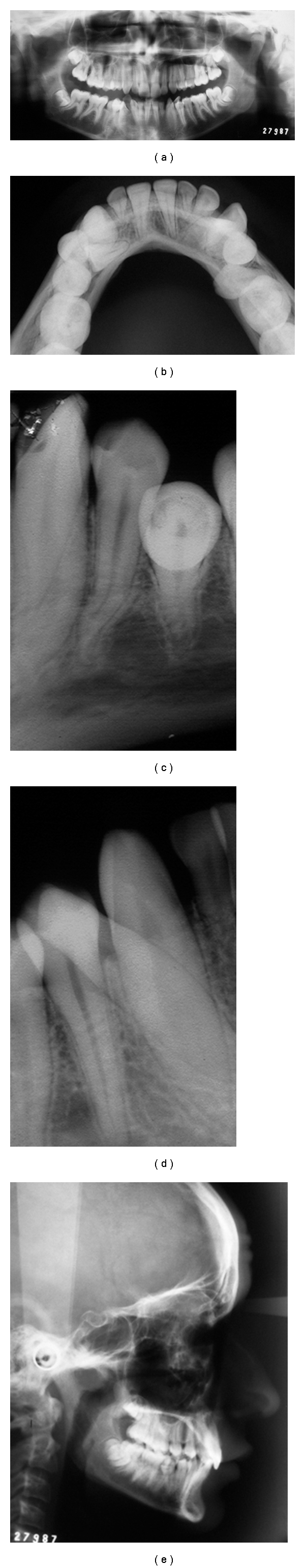


On radiographic examination, the presence of third molar germs in all quadrants was observed. Ectopic eruption of the permanent mandibular right lateral incisor and partial transposition of permanent mandibular left lateral incisor and left canine had been also diagnosed since the apices of the lateral incisors and canines were correctly positioned, but the coronal position was altered due to inclination of the lateral incisor (Figure 3). 

Both patient and caregiver were instructed about the current situation of the case. Also, they were informed on the need for corrective orthodontic treatment in association with extraction of deciduous teeth and premolars (maxillary and mandibular ones) for aligning and levelling the dental arches as well as for correcting both ectopia and Class II division 2 relationship. However, they decided for no orthodontic treatment.

## 3. Discussion

**Figure 4:**
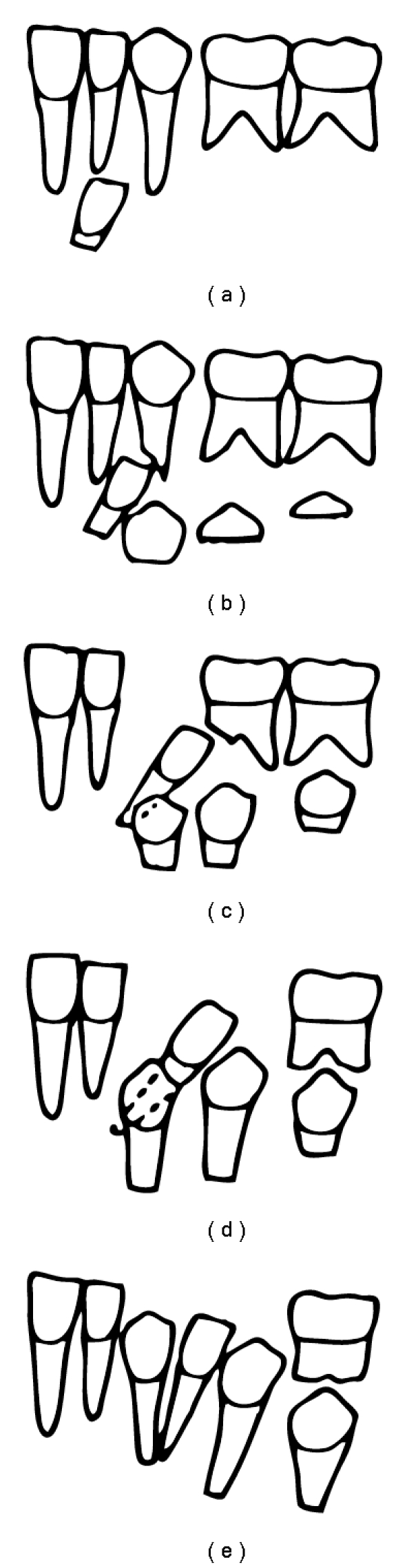
Possible evolution of a nontreated ectopic eruption of a mandibular lateral incisor: early exfoliation of primary canines and first molars and partial transposition.

Dental ectopia is characterised by abnormal or even aberrant eruption of one or more teeth, thus resulting in root absorption of the adjacent teeth. Transposition of the teeth is the more severe effect, which consists of positional switch between two adjacent teeth or eruption of one tooth into normal position already occupied by another nonadjacent tooth [5, 16, 20]. As can be seen in Figure 4, such a transposition can be complete, when crowns and roots are found to be transposed and paralleled, or partial, when crowns are found to be transposed and root apices are in relatively normal positions [1, 20, 21]. The aetiological possibilities may involve genetic aspects and physical obstacles. Regarding the former, no past history of ectopia was found in the patient's family whereas the Meckel's cartilage remaining in the alveolar region of the canines [22], which may obstruct physically the normal eruptive pathway of the tooth, is another possible aetiology.

The present case was particularly interesting because it reported two conditions, namely, ectopia of the mandibular lateral incisor on the right side and partial transposition of the mandibular lateral incisor on the left side. The differential diagnosis was achieved by localising the root apices of the left teeth, which had virtually normal proximal positioning despite the transposed crowns. 

In general, ectopic lateral incisors display both distal inclination and marked rotation [7, 13, 17]. In the present case, the left mandibular lateral incisor had 90° rotation whereas the right mandibular lateral incisor had 30° rotation, with distal inclinations of 30° and 20°, respectively, in relation to the occlusal plane. Deviation in the eruption axis of lateral incisors provokes prolonged retention of the deciduous lateral incisors and even the canines [4, 13, 18], as could be seen in the present case.

When ectopia is detected early, the ectopic mandibular lateral incisors can be corrected by extracting the mandibular deciduous canines and vertically positioning the affected teeth. This orthodontic movement should be retained as long as possible since tooth tends to retake their wrong position. Consequently, transposition between ectopic lateral incisor and the developing canine germ is prevented from occurring [7, 9, 15, 17, 18, 23, 24]. Radiographic examination is recommended for 6–8-year-old children so that the dental malpositioning can be precociously diagnosed [20].

If treatment is delayed, as the case presented herein, there is general agreement that transposition should not be corrected in the mandibular arch because the buccal-lingual space is not enough for accommodating tooth movements, which might provoke root interference resulting in root absorption as well as damage to the supporting tissues [9, 10, 18, 20, 25–27]. Treatment interventions include either alignment of the teeth in their transposed order [18, 25–27] or extraction of the ectopic lateral incisor [9, 14, 15, 27], which can be prosthetically replaced if space still exists [15].

Due to the importance of the early diagnosis in those cases of eruptive alteration, dentists should take into account the followup of both tooth eruption and formation of permanent dentition so that any change in the normal dental development can be diagnosed and readily treated.
